# Regional high iron deposition on quantitative susceptibility mapping correlates with cognitive decline in type 2 diabetes mellitus

**DOI:** 10.3389/fnins.2023.1061156

**Published:** 2023-01-30

**Authors:** Rui Hu, Bingbing Gao, Shiyun Tian, Yangyingqiu Liu, Yuhan Jiang, Wanyao Li, Yuan Li, Qingwei Song, Weiwei Wang, Yanwei Miao

**Affiliations:** Department of Radiology, The First Affiliated Hospital of Dalian Medical University, Dalian, China

**Keywords:** type 2 diabetes mellitus, quantitative susceptibility mapping, magnetic sensitivity value, deep gray nuclei, volume, heterogeneity, brain iron deposition

## Abstract

**Objective:**

To quantitatively evaluate the iron deposition and volume changes in deep gray nuclei according to threshold-method of quantitative susceptibility mapping (QSM) acquired by strategically acquired gradient echo (STAGE) sequence, and to analyze the correlation between the magnetic susceptibility values (MSV) and cognitive scores in type 2 diabetes mellitus (T2DM) patients.

**Methods:**

Twenty-nine patients with T2DM and 24 healthy controls (HC) matched by age and gender were recruited in this prospective study. QSM images were used to evaluate whole-structural volumes (V_wh_), regional magnetic susceptibility values (MSV_RII_), and volumes (V_RII_) in high-iron regions in nine gray nuclei. All QSM data were compared between groups. Receiver operating characteristic (ROC) analysis was used to assess the discriminating ability between groups. The predictive model from single and combined QSM parameters was also established using logistic regression analysis. The correlation between MSV_RII_ and cognitive scores was further analyzed. Multiple comparisons of all statistical values were corrected by false discovery rate (FDR). A statistically significant *P*-value was set at 0.05.

**Results:**

Compared with HC group, the MSV_RII_ of all gray matter nuclei in T2DM were increased by 5.1–14.8%, with significant differences found in bilateral head of caudate nucleus (HCN), right putamen (PUT), right globus pallidus (GP), and left dentate nucleus (DN) (*P* < 0.05). The V_wh_ of most gray nucleus in T2DM group were decreased by 1.5–16.9% except bilateral subthalamic nucleus (STN). Significant differences were found in bilateral HCN, bilateral red nucleus (RN), and bilateral substantia nigra (SN) (*P* < 0.05). V_RII_ was increased in bilateral GP, bilateral PUT (*P* < 0.05). V_RII_/V_wh_ was also increased in bilateral GP, bilateral PUT, bilateral SN, left HCN and right STN (*P* < 0.05). Compared with the single QSM parameter, the combined parameter showed the largest area under curve (AUC) of 0.86, with a sensitivity of 87.5% and specificity of 75.9%. The MSV_RII_ in the right GP was strongly associated with List A Long-delay free recall (List A LDFR) scores (*r* = −0.590, *P* = 0.009).

**Conclusion:**

In T2DM patients, excessive and heterogeneous iron deposition as well as volume loss occurs in deep gray nuclei. The MSV in high iron regions can better evaluate the distribution of iron, which is related to the decline of cognitive function.

## 1. Introduction

The prevalence of type 2 diabetes mellitus (T2DM) has increased rapidly and T2DM has become one of the most significant metabolic diseases and major worldwide health problems. Studies have shown that T2DM patients are often accompanied by central nervous system damage, including cognitive impairment (Pasquier et al., 2006; McCrimmon et al., 2012; Degen et al., 2016).

Brain iron deposition has been identified to be associated with cognitive decline in various neurodegenerative diseases, such as Alzheimer’s disease (AD) and Parkinson’s disease (PD) (Du et al., 2018; Ayton et al., 2020; Thomas et al., 2020). A few T2DM studies (Yang et al., 2018; Li et al., 2020a,b) have found increased brain iron deposits in gray nuclei, which is related to cognitive decline.

In recent years, Quantitative susceptibility mapping (QSM) has been regarded as the preferred method for quantitative analysis of brain iron because it can quantify the magnetic susceptibility of tissue accurately (Langkammer et al., 2012; Yan et al., 2018). There are many approaches to obtain QSM now, and among them, strategically acquired gradient echo (STAGE) is a rapid multi-contrast and multi-parametric imaging sequence that provides multiple qualitative and quantitative images including QSM. Meanwhile, the QSM reconstruction from STAGE data helps to improve the signal-to-noise ratio and yields fewer artifacts (Gharabaghi et al., 2020). Previous studies have found that STAGE provides a reproducible standardization for iron content measurement (Chen et al., 2018; He et al., 2022).

Post-mortem studies showed that there was a significant correlation between magnetic susceptibility values (MSV) in QSM and histochemical iron contents in brain tissues (Langkammer et al., 2012; Sun et al., 2015; Hametner et al., 2018; Lee et al., 2018; Lewis et al., 2018; Wang et al., 2020). The magnetic susceptibility of biological tissue reflects its magnetization under external magnetic fields, its value can describe the compositional changes in tissues (Guo et al., 2018). However, the MSV measurement methods used in different studies are inconsistent. In a T2DM study, Li et al. (2020b) detected more iron overloading nuclei using voxel-based analysis than region of interest (ROI)-based analysis (Li et al., 2020a), indicating that voxel-based analysis is more sensitive in brain iron detection. However, their measurements are based on the whole structure, which cannot reflect the uneven distribution of iron in the substructures well. More studies have found heterogeneity in brain iron deposition, even in the same nucleus (Sakurai et al., 2010; Wang et al., 2012; Han et al., 2013; Ghassaban et al., 2019; Lee and Lee, 2019). The method of whole-structure-iron measurement is difficult to display regional iron changes in nucleus. ROI-based regional high iron analysis may have a higher diagnostic value than whole structural iron analysis, and reduces the possible error of manually ROI drawing (Wang et al., 2012, 2016) greatly. Previous studies (Ghassaban et al., 2019) have reported abnormal iron deposition in the substantia nigra (SN), especially in regional high iron areas, which can be used as a biomarker to distinguish patients with PD from healthy controls (HC) and to assess the severity of the disease.

However, the previous T2DM studies about brain iron loads (Yang et al., 2018; Li et al., 2020a,b) were analyzed based on the whole nuclei rather than the regional ones. To our knowledge, there is no study on the correlation between the heterogeneity distribution of brain iron and cognitive state in T2DM patients up to now.

Based on the above, we intend to quantitatively analyze the whole-structural volumes, regional MSV and volumes in various nuclei, and to explore the relationship between these parameters and cognitive state in T2DM patients.

## 2. Materials and methods

### 2.1. Participants

This prospective study was approved by the local ethics committee of the First Affiliated Hospital of Dalian Medical University. All participants were recruited from the local community with recruitment advertisements. Informed consents were obtained from all participants before the study.

The clinical diagnostic criteria for T2DM were in line with the recommendations of the American Diabetes Association (ADA) in 2014 (Chung et al., 2014). All patients were right-handed and had no contraindications for MRI scanning. Exclusion criteria included: (a) seriously internal medicine emergency; (b) history of drug abuse or dependence, alcoholism, etc.; (c) history of current or previous neuropsychiatric illness, family history of psychiatric illness; (d) combined with severe brain diseases, such as massive cerebral infarction, cerebral hemorrhage, tumors, trauma, etc.; (e) current or previous history of severe physical diseases; (f) history of craniocerebral surgery.

Thirty-four patients with T2DM were prospectively recruited from July 2019 to November 2019 in this study. Three patients with poor MRI image quality and two patients with motion artifacts were excluded. At last, 29 T2DM patients (15 males; 59.83 ± 9.38 years) were enrolled in the analysis. In addition, we recruited 24 healthy volunteers for controls (HC, 9 males; 56.83 ± 7.29 years) during the corresponding period. They were all right-handed and matched with T2DM groups in terms of age, gender, and education degrees. The exclusion criteria for HC were: (a) history of diabetes and hypertension; (b) traumatic brain injury or structural abnormality; (c) other exclusion criteria are the same as those of T2DM.

### 2.2. Clinical characteristics

Clinical data including age, gender, body mass index (BMI), and education years were collected from all subjects. Blood biochemical tests which include fasting plasma glucose (FG), glycated hemoglobin (HbA1c) and hemoglobin (Hb) were recorded in all participants before MR data acquisition within 1 week.

### 2.3. Neurocognitive assessments

Neurocognitive assessments were performed before the MR scan within 1 week, including mini-mental state examination (MMSE), Montreal cognitive assessment (MoCA), and California verbal learning test (CVLT). CVLT can be used to check the verbal learning and memory processing function of patients. The primary measures include recall after the first trial (CVLT trial 1 Sum), the fifth trial (CVLT trial 5 Sum), the sum across the five trials (CVLT trials 1–5 Sum), List A Short-delay free recall (List A SDFR), List A Short-delay cued recall (List A SDCR), List A Long-delay free recall (List A LDFR), and List A Long-delay cued recall (List A LDCR).

### 2.4. MR image acquisition

All of the participants underwent routine sequences and STAGE on a 3.0T MR scanner (Ingenia CX, Philips Healthcare, Best, Netherlands) equipped with a 32-channel phased-array head coil. The STAGE parameters were: flip angle (FA) = 6°/24°, echo time (TE_1_) = 7.5 ms/8.75 ms, TE_2_ = 17.5 ms/18.75 ms, repetition time (TR) = 30 ms, field of view (FOV) = 256 mm × 256 mm × 128 mm, matrix = 384 × 191 × 64, voxel = 0.87 mm × 1.34 mm × 2 mm. T1WI: FA = 75°, TE = 6.9 ms, TR = 297 ms, FOV = 230 mm × 183 mm, matrix = 232 × 178, NEX = 1, slice thickness = 3.0 mm, slice gap = 0 mm. T2WI: TE = 13.81 ms, and TR = 1,186 ms, FOV = 230 mm × 183 mm, matrix = 256 × 164, NEX = 1, slice thickness = 3.0 mm, slice gap = 0 mm.

### 2.5. QSM processing and measurement

Raw STAGE data was transmitted in Digital Imaging and Communications in Medicine (DICOM) format to a personal computer, and processed according to an established STAGE pipeline in SPIN software (SpinTech, Inc., Bingham Farms, MI, USA). Multiple qualitative and quantitative images were further obtained in STAGE software, such as T1WE, T1 MAP, PD MAP, SWI, tSWI, R2*, and QSM image, and then QSM images were selected to measure MSV.

**FIGURE 1:**
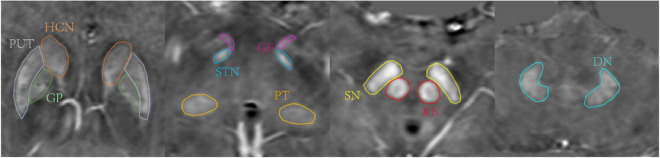
Region of interests (ROIs) traced on quantitative susceptibility mapping (QSM) of a 47-year-old male. Structures include head of caudate nucleus (HCN, brown), globus pallidus (GP, green), putamen (PUT, gray), pulvinar thalamus (PT, orange), subthalamic nucleus (STN, blue), red nucleus (RN, red), substantia nigra (substantia nigra (SN), yellow), dentate nucleus (DN, pale blue), gyri fasciolaris (GF, pink).

Two neuroradiologists with more than 7 years of work experience manually drew 3D whole-structural ROIs along the border of bilateral gray nuclei, including head of caudate nucleus (HCN), globus pallidus (GP), putamen (PUT), pulvinar thalamus (PT), subthalamic nucleus (STN), red nucleus (RN), substantia nigra (SN), dentate nucleus (DN), and gyri fasciolaris (GF), as shown in Figure 1. The boundary of gray nuclei was defined referring to T1WE and tSWI images. The ventricle and vessel were avoided when drawing the ROIs.

**FIGURE 2:**
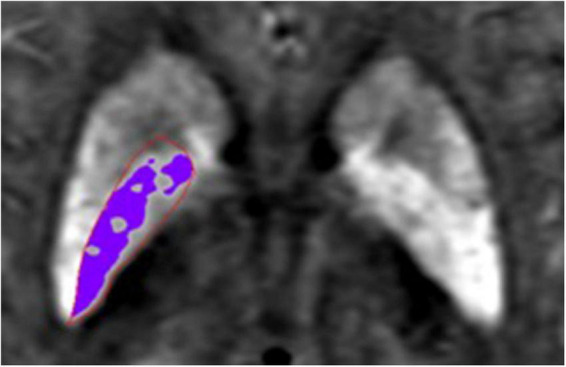
Schematic diagram of R_II_ area. The area in the red region of interest (ROI) is globus pallidus (GP), the purple area represents the iron concentration is larger than the threshold in the GP.

An MSV threshold from the HC group was calculated to define the boundary of high iron content (R_II_, Figure 2) which could evaluate the iron content in the nucleus more accurately. Mean plus two standard deviations were used to calculate the MSV threshold of each nucleus (Table 1) according to the study of Ghassaban et al. (2019). R_II_ region is the area of the structure where the iron concentration is larger than the threshold. The MSV of R_II_ region (MSV_RII_), volumes of R_II_ region (V_RII_), and whole nucleus (V_wh_) were measured, and further V_RII_/V_wh_ were calculated.

**TABLE 1:** Threshold of magnetic susceptibility values (MSV).

Region	Mean	SD	Threshold
HCN-right	42.47	7.81	58.09
HCN-left	38.75	7.66	54.07
PUT-right	42.52	9.06	60.64
PUT-left	46.09	10.71	67.51
GP-right	115.99	25.24	166.47
GP-left	115.36	26.55	168.46
SN-right	110	17.76	145.52
SN-left	115.97	19.48	154.93
RN-right	104.05	23.85	151.75
RN-left	96.15	25.6	147.35
DN-right	75.67	20.41	116.49
DN-left	75.69	23.61	122.91
PT-right	31.76	4.73	41.22
PT-left	29.61	5.24	40.09
STN-right	72.4	19.72	111.84
STN-left	76.75	18.37	113.49
GF-right	54.23	6.01	66.25
GF-left	48.5	7.92	64.34

Mean, mean value of MSV; SD, standard deviations; Threshold, Mean + 2SD; HCN, head of caudate nucleus; GP, globus pallidus; PUT, putamen; PT, pulvinar thalamus; STN, subthalamic nucleus; RN, red nucleus; SN, substantia nigra; DN, dentate nucleus; GF, gyri fasciolaris.

### 2.6. Statistical analysis

The statistical package for social science (SPSS) version 25.0 and R version 4.1.2 were used to analyze the data. In order to determine the reliability of inter-observer relationships, the intraclass correlation coefficient (ICC) test was conducted. Independent samples *t*-test (for normally distributed data) or Mann–Whitney U test (for non-normally distributed data) or Chi-square test (enumeration data) was used to compare the MSV_RII_, V_RII_, V_wh_, V_RII_/V_wh_ of gray matter nucleus, clinical data, laboratory examination indexes and cognitive scores between T2DM and HC group. Pearson correlation analysis (for normally distributed data) or Spearman correlation analysis (for non-normally distributed data) was used to analyze the correlation between MSV_RII_ with significant differences and cognitive scores. Receiver operating characteristic (ROC) test was used to assess the ability of MSV_RII_, V_RII_, and V_wh_ to discriminate differences between groups, the predictive model was established by logistic regression analysis, and the difference in efficacy between single parameter and the combined diagnosis was compared with Delong test. Multiple comparisons were corrected by false discovery rate (FDR) correction (Benjamini–Hochberg method). A statistically significant *P-*value was set at 0.05.

## 3. Results

### 3.1. Subject characteristics

A summary of all demographic, clinical, and cognitive characteristics can be found in Table 2. There were no significant differences in gender, age, education years, BMI, Hb, CVLT, or MMSE between groups (*P* > 0.05). The poorer MoCA performance was found in T2DM patients (*P* < 0.001).

**TABLE 2:** Demographic, clinical data, and cognitive scores.

Characteristics	T2DM (*n* = 29)	HC (*n* = 24)	*x*^2/^*t*/*z*	*P*-value
Gender (M/F)	15/14	9/15	1.072	0.300
Age^a^	59.83 ± 9.38	56.83 ± 7.29	1.276	0.208
Education years*^b^*	12.00 (9.00–15.50)	12.00 (9.00–15.00)	-1.215	0.224
BMI^a^	25.31 ± 2.30	24.03 ± 2.54	-1.922	0.060
FG^a^	8.21 ± 2.69	4.62 ± 0.42	-4.743	< **0**.001*
HbA1c^a^	7.39 ± 1.32	5.35 ± 0.19	-5.484	< **0**.001*
Hb^a^	144.07 ± 15.64	140.13 ± 14.12	-0.955	0.344
CVLT trials 1 Sum^a^	4.86 ± 1.57	5.15 ± 1.57	0.555	0.582
CVLT trials 5 Sum^a^	9.83 ± 1.79	10.31 ± 2.25	0.741	0.463
CVLT trials 1–5 Sum^a^	39.97 ± 7.01	42.00 ± 7.33	0.858	0.396
List A SDFR^a^	8.38 ± 1.97	8.92 ± 1.19	0.919	0.364
List A SDCR^a^	9.72 ± 1.89	10.00 ± 1.58	0.459	0.649
List A LDFR^b^	9.00 (8.00–10.00)	9.00 (8.00–10.50)	-0.263	0.792
List A LDCR^a^	9.41 ± 2.53	9.54 ± 2.07	0.156	0.877
MMSE^b^	28.00 (27.50–29.50)	29.00 (28.00–30.00)	-0.898	0.369
MCoA^b^	26.00 (24.00–27.50)	28.00 (27.00–29.00)	-3.731	< **0**.001*

^a^Data was expressed as mean value ± standard deviations.

^b^Data was expressed as median (1/4–3/4).

*P-values in bold show significant differences (*P < 0.05). BMI, body mass index; FG, fasting plasma glucose; HbA1c, glycated hemoglobin; Hb, hemoglobin; List A SDFR, List A Short-delay free recall; List A SDCR, List A Short-delay cued recall; List A LDFR, List A Long-delay free recall; List A LDCR, List A Long-delay cued recall.

### 3.2. MSV_RII_ and volume changes between groups

**FIGURE 3:**
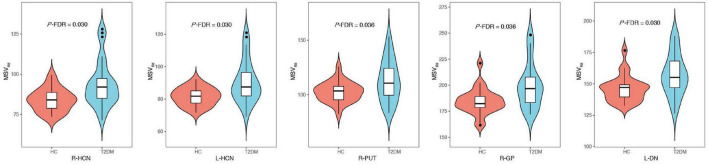
Comparisons of gray nucleus magnetic susceptibility values (MSV_RII_) with significant differences between type 2 diabetes mellitus (T2DM) group and healthy controls (HC) group, there were significant differences in bilateral head of caudate nucleus (HCN), **right** PUT, **right** globus pallidus (GP) and **left** dentate nucleus (DN) (*P*-FDR < 0.05).

An ICC analysis revealed excellent inter-rater consistency across all measured data (0.802–0.965). Compared with the HC group, the MSV_RII_ of all gray nuclei in the T2DM group were increased by 5.1–14.8%, and there were significant differences in bilateral HCN, right PUT, right GP, and left DN (*P* < 0.05, Table 3 and Figure 3).

**TABLE 3:** Comparison of magnetic susceptibility values (MSV_RII_) and V_wh_ between type 2 diabetes mellitus (T2DM) and healthy controls (HC).

Region		MSV_RII_ (ppb)			V_wh_ (mm^3^)		
		**T2DM**	**HC**	***P*-FDR**	**T2DM**	**HC**	***P*-FDR**
HCN	Right	92.02 (84.45–99.47)	83.99 (78.57–88.74)	**0.030** ^b^	1,991.11 (1,805.77–2,117.33)	2,197.90 (2,127.33–2,383.55)	**0.000** ^b^
	Left	87.53 (81.88–98.08)	81.81 (77.38–85.86)	**0.030** ^b^	2,012.66 ± 363.49	2,385.32 ± 321.66	**0.000** ^a^
PUT	Right	112.83 ± 18.96	102.63 ± 9.15	**0.036** ^a^	2,962.27 ± 518.20	3,066.73 ± 454.36	0.570^a^
	Left	115.67 ± 23.37	104.58 ± 6.91	0.06^a^	2,793.17 ± 498.74	2,855.03 ± 437.62	0.716^a^
GP	Right	196.66 (182.06–209.51)	182.20 (177.61–189.22)	**0.036** ^b^	2,166.10 ± 291.81	2,267.44 ± 253.08	0.282^a^
	Left	191.64 (182.86–213.93)	182.44 (176.93–190.81)	0.069^b^	1,978.79 ± 308.65	2,131.83 ± 362.72	0.264^a^
SN	Right	195.36 (183.52–209.20)	187.46 (182.61–194.67)	0.102^b^	713.84 ± 98.36	837.11 ± 157.65	**0.007** ^a^
	Left	193.40 (181.89–219.90)	185.91 (175.92–194.06)	0.090^b^	701.46 ± 116.12	789.69 ± 140.28	**0.045** ^a^
RN	Right	178.53 (169.86–195.55)	169.76 (159.88–181.60)	0.083^b^	261.33 (246.22–294.22)	331.16 (294.66–373.77)	**0.006** ^b^
	Left	178.88 (162.01–190.44)	169.94 (159.37–183.02)	0.174^b^	257.78 (243.56–294.22)	329.71 (266.89–359.33)	**0.007** ^b^
DN	Right	155.11 ± 16.88	147.64 ± 13.15	0.107^a^	1,084.08 ± 217.97	1,173.47 ± 265.71	0.282^a^
	Left	155.11 (146.30–169.29)	147.05 (136.11–149.35)	**0.030** ^b^	1,071.82 ± 212.19	1,179.93 ± 287.44	0.274^a^
PT	Right	58.11 (50.43–66.65)	53.54 (49.18–56.01)	0.140^b^	822.65 ± 211.71	904.69 ± 222.07	0.282^a^
	Left	54.14 (49.96–67.68)	51.70 (47.21–54.88)	0.095^b^	824.43 ± 181.48	879.53 ± 267.92	0.523^a^
STN	Right	146.64 (132.28–157.03)	133.81 (122.80–150.02)	0.095^b^	56.83 ± 13.81	55.73 ± 17.68	0.848^a^
	Left	146.58 (127.52–155.63)	135.58 (124.26–148.59)	0.125^b^	61.64 ± 18.56	54.47 ± 17.36	0.282^a^
GF	Right	91.15 (79.15–102.47)	83.39 (76.28–91.46)	0.102^b^	31.26 ± 7.19	32.82 ± 10.51	0.650^a^
	Left	84.17 (77.57–105.78)	83.46 (73.77–92.04)	0.167^b^	35.34 ± 9.05	35.88 ± 15.46	0.881^a^

^a^Data was expressed as mean value ± standard deviations.

^b^Data was expressed as median (1/4–3/4).

HCN, head of caudate nucleus; GP, globus pallidus; PUT, putamen; PT, pulvinar thalamus; STN, subthalamic nucleus; RN, red nucleus; SN, substantia nigra; DN, dentate nucleus; GF, gyri fasciolaris. *P*-FDR values in bold show significant differences between the means after FDR correction (*P*-FDR < 0.05).

The V_RII_ in bilateral GP and PUT were increased in the T2DM groups compared to HCs (*P* < 0.05, Table 4). Except for bilateral STN, the V_wh_ of all gray matter nuclei in the T2DM group were decreased by 1.5–16.9%. Significant V_wh_ differences were found in bilateral HCN, bilateral RN and bilateral SN, (*P* < 0.05, Table 3).

**TABLE 4:** Comparison of V_RII_ and V_RII_/V_wh_ between type 2 diabetes mellitus (T2DM) and healthy controls (HC).

		V_RII_ (mm^3^)			V_RII_/V_wh_		
**Hemisphere**		**T2DM**	**HC**	***P*-FDR**	**T2DM**	**HC**	***P*-FDR**
HCN	Right	639.72 ± 331.78	546.11 ± 259.84	0.349^a^	0.33 ± 0.16	0.25 ± 0.12	0.065^a^
	Left	606.56 ± 343.93	512.26 ± 210.97	0.349^a^	0.30 ± 0.15	0.22 ± 0.09	**0.039^a^**
PUT	Right	1,015.69 ± 578.19	641.32 ± 309.75	**0.036** ^a^	0.34 ± 0.18	0.21 ± 0.10	**0.018** ^a^
	Left	989.58 ± 549.25	669.05 ± 277.57	**0.040** ^a^	0.36 ± 0.19	0.24 ± 0.09	**0.018** ^a^
GP	Right	575.48 ± 390.27	306.61 ± 222.30	**0.036** ^a^	0.26 ± 0.17	0.13 ± 0.09	**0.018** ^a^
	Left	488.31 ± 336.63	283.26 ± 201.66	**0.040** ^a^	0.24 ± 0.17	0.13 ± 0.09	**0.018** ^a^
SN	Right	246.22 (120.89–335.11)	196.44 (138.22–221.97)	0.296^b^	0.34 ± 0.15	0.24 ± 0.09	**0.018** ^a^
	Left	252.44 ± 146.98	190.85 ± 82.60	0.162^a^	0.35 ± 0.19	0.24 ± 0.10	**0.039** ^a^
RN	Right	76.45 (36.44–132.88)	46.67 (15.60–94.58)	0.296^b^	0.31 (0.14–0.50)	0.13 (0.04–0.32)	0.065^b^
	Left	87.11 (23.55–123.55)	42.64 (14.98–103.38)	0.349^b^	0.34 (0.08–0.49)	0.12 (0.06–0.31)	0.065^b^
DN	Right	297.78 (185.33–411.11)	244.42 (91.62–325.67)	0.349^b^	0.28 ± 0.15	0.21 ± 0.12	0.093^a^
	Left	318.22 (202.22–438.22)	267.16 (92.89–371.55)	0.349^b^	0.32 (0.18–0.40)	0.23 (0.09–0.29)	0.054^b^
PT	Right	337.90 ± 233.74	281.80 ± 185.69	0.388^a^	0.39 ± 0.21	0.30 ± 0.17	0.126^a^
	Left	312.89 (92.89–497.33)	220.95 (81.62–283.30)	0.365^b^	0.36 ± 0.23	0.25 ± 0.15	0.065^a^
STN	Right	24.00 (18.22–29.78)	13.78 (8.93–20.24)	0.050^b^	0.43 ± 0.20	0.30 ± 0.17	**0.043** ^a^
	Left	26.00 ± 18.02	18.56 ± 9.77	0.162^a^	0.40 ± 0.23	0.35 ± 0.16	0.346^a^
GF	Right	13.95 ± 8.27	12.69 ± 8.16	0.604^a^	0.44 ± 0.24	0.40 ± 0.23	0.558^a^
	Left	13.33 (6.22–20.44)	12.62 (4.76–19.55)	0.604^b^	0.42 ± 0.24	0.36 ± 0.18	0.346^a^

^a^Data was expressed as mean value ± standard deviations.

^b^Data was expressed as median (1/4–3/4).

HCN, head of caudate nucleus; GP, globus pallidus; PUT, putamen; PT, pulvinar thalamus; STN, subthalamic nucleus; RN, red nucleus; SN, substantia nigra; DN, dentate nucleus; GF, gyri fasciolaris. *P*-FDR values in bold show significant differences between the means after FDR correction (*P*-FDR < 0.05).

The V_RII_/V_wh_ of all gray matter nuclei in the T2DM group were larger than HC, and significant differences were found in bilateral GP, bilateral PUT, bilateral SN and left HCN, right STN (*P* < 0.05, Table 4).

**FIGURE 4:**
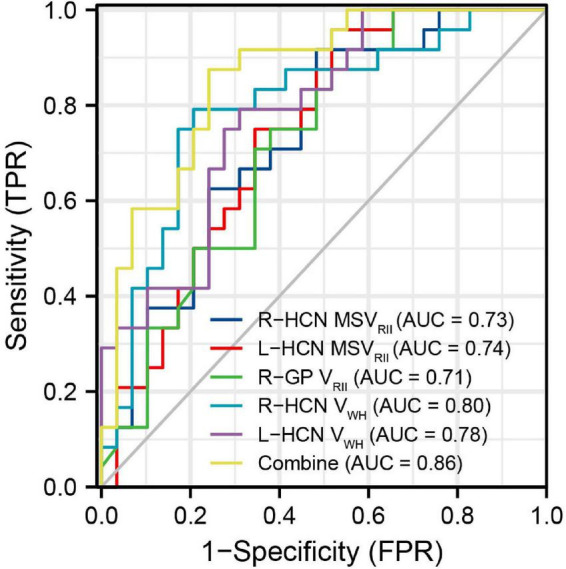
The receiver operating characteristic (ROC) map of conjoint variable analysis composed with five meaningful parameters (combine) had the largest area under curve (AUC) of 0.86.

Receiver operating characteristic analysis of MSV_RII_, V_RII_, and V_wh_ were summarized in Table 5. The largest area under curve (AUC) of 0.86, the sensitivity of 87.5% and the specificity was 75.9% were obtained when five parameters of bilateral HCN-V_wh_, bilateral HCN-MSV_RII_ and right GP-V_RII_ were combined (Figure 4). Delong test showed that the performance of conjoint diagnosis was significantly improved compared with a single parameter (*P* < 0.05).

**TABLE 5:** Receiver operating characteristic (ROC) analysis of magnetic susceptibility values (MSV_RII_), V_RII_, and V_wh_ between type 2 diabetes mellitus (T2DM) and healthy controls (HC).

Predictor variable	AUC	Cut-off	Sensitivity	Specificity
R-HCN-MSV_RII_	0.73	91.94	0.92	0.52
L-HCN-MSV_RII_	0.74	89.41	0.96	0.48
R-PUT-MSV_RII_	0.66	109.46	0.83	0.55
R-GP-MSV_RII_	0.71	195.67	0.88	0.62
L-DN-MSV_RII_	0.73	150.76	0.83	0.66
R-PUT-V_RII_	0.70	915.56	0.88	0.66
L-PUT-V_RII_	0.68	903.11	0.79	0.62
R-GP-V_RII_	0.714	445.78	0.75	0.62
L-GP-V_RII_	0.70	274.23	0.67	0.72
R-HCN-V_wh_	0.80	2,121.78	0.79	0.79
L-HCN-V_wh_	0.78	2,156.49	0.79	0.69
R-SN-V_wh_	0.75	771.25	0.71	0.76
L-SN-V_wh_	0.70	723.58	0.71	0.66
R-RN-V_wh_	0.76	292.89	0.79	0.76
L-RN-V_wh_	0.75	285.33	0.75	0.76
Combine	0.86	-	0.88	0.76

R-HCN, right head of caudate nucleus; L-HCN, left right head of caudate nucleus; R-PUT, right putamen; L-PUT, left putamen; R-GP, right globus pallidus; L-GP, left globus pallidus; L-DN, left dentate nucleus; R-RN, right red nucleus; L-RN, left red nucleus; R-SN, right substantia nigra; L-SN, left substantia nigra; Combine, five meaningful parameters of bilateral HCN-V_wh_, bilateral HCN-MSV_RII_ and right GP-V_RII_ were combined.

### 3.3. Correlation between MSV_RII_ and cognitive scores in T2DM patients

**FIGURE 5:**
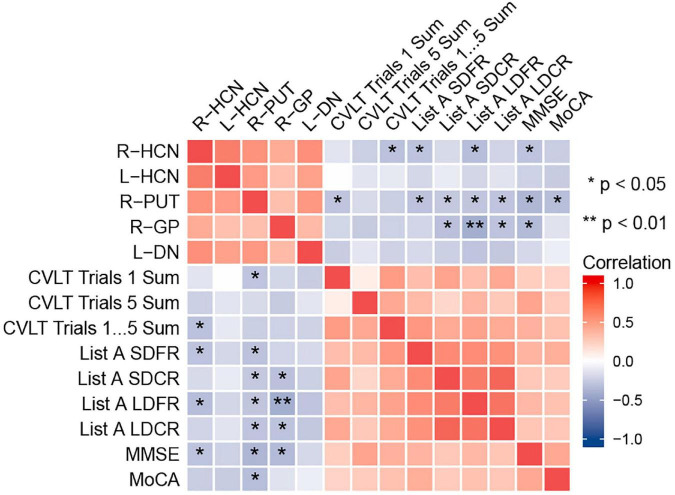
Heat map showing the correlation analysis between gray nucleus magnetic susceptibility values (MSV_RII_) with significant differences and cognitive scores in type 2 diabetes mellitus (T2DM) group. Color bar on the right side displays the value of the correlation coefficient (higher from –1 to 1, and from blue to red). *P < 0.05, **P < 0.01. List A SDFR, List A Short-delay free recall; List A SDCR, List A Short-delay cued recall; List A LDFR, List A Long-delay free recall; List A LDCR, List A Long-delay cued recall.

**FIGURE 6:**
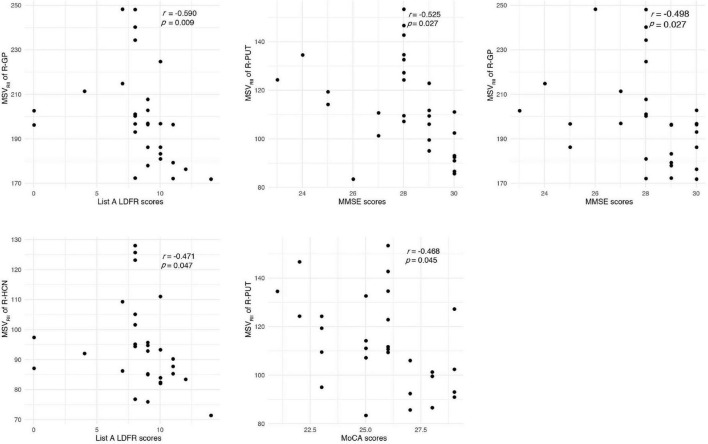
Correlations between magnetic susceptibility values (MSV_RII_) and cognitive score. The correlation coefficient lager than –0.45 were represented by scatter plots.

The correlation between the MSV_RII_ of gray matter nuclei with significant differences and cognitive scores in T2DM group were shown in Figure 5. The MSV_RII_ in the right GP (*r* = −0.590, *P* = 0.009), right HCN (*r* = −0.471, *P* = 0.047), and right PUT (*r* = −0.411, *P* = 0.045) were negatively correlated with List A LDFR. The MSV_RII_ in the right PUT (*r* = −0.525, *P* = 0.027), right GP (*r* = −0.498, *P* = 0.027), and right HCN (*r* = −0.427, *P* = 0.047) were negatively correlated with MMSE. The MSV_RII_ in the right PUT (*r* = −0.468, *P* = 0.045) was negatively correlated with MoCA. The MSV_RII_ in the right GP (*r* = −0.449, *P* = 0.045) and right PUT (*r* = −0.393, *P* = 0.045) were negatively correlated with List A SDCR. The MSV_RII_ in the right HCN (*r* = −0.441, *P* = 0.047) was negatively correlated with CVLT trials 1–5 Sum. The MSV_RII_ in the right PUT (*r* = −0.434, *P* = 0.045), right HCN (*r* = −0.429, *P* = 0.047) were negatively correlated with List A SDFR. The MSV_RII_ in the right GP (*r* = −0.430, *P* = 0.045), right PUT (*r* = −0.421, *P* = 0.045) were negatively correlated with List A LDCR. The MSV_RII_ in the right PUT (*r* = −0.420, *P* = 0.045) was negatively correlated with CVLT trials 1 Sum. Among these, the correlation coefficient lager than −0.45 were represented by scatter plots (Figure 6).

However, no correlation was found between MSV_RII_ of gray matter nuclei with significant differences and V_wh_ in the T2DM group (*P* > 0.05).

## 4. Discussion

Iron deposition has been found associated with oxidative phosphorylation, while increasing levels of oxidative stress may add the risk of T2DM (Rajpathak et al., 2009). Iron overload could generate reactive oxygen species, thereby destroying DNA, eventually leading to end in neuronal cell death and memory decline (Rouault, 2001; Poon et al., 2004; Melis et al., 2013; Cozzi et al., 2019). Previous studies have shown that a history of T2DM could increase iron deposition in some structures of deep gray matter in elderly individuals (Li et al., 2021). The elevated glucose level may aggravate the iron deposition, and iron accumulation produce damage to brain cells resulting in insulin resistance. The relationship between them seems to be bidirectional (Biessels et al., 1998; Fernández-Real et al., 2002).

With QSM, increased water has a limited effect on relaxation values, so the confounding effect can be avoided, this technique is highly sensitive and represents the most accurate way to measure brain iron levels (Yan et al., 2018). Non-invasive quantitative analysis of brain iron deposition can be achieved by MSV measurement based QSM. Because of the lower confounding effect of myelin and negligible contribution from other paramagnetic materials, QSM has proven to be an accurate method of measuring iron content in deep gray matter structures (Langkammer et al., 2012). In post-mortem studies, QSM contrast is significantly correlated with histochemical measurements of iron in these regions, demonstrating its accuracy at identifying iron deposition (Langkammer et al., 2012; Sun et al., 2015; Hametner et al., 2018; Lee et al., 2018; Lewis et al., 2018; Wang et al., 2020).

The precise mechanisms underlying the higher iron concentration in T2DM are not understood yet. This study combined R_II_ iron content and volume changes of gray matter nuclei in T2DM patients. The differences patterns were explored not only from the perspective of regional high iron and volume, but also from the perspective of whole region volume, and the relationship between abnormal R_II_ iron accumulation and cognitive decline were also evaluated. In this study, it was found that the MSV_RII_, V_RII_, and V_RII_/V_wh_ of all gray matter nuclei in T2DM group were increased, but the V_wh_ of most gray matter nuclei in T2DM group was decreased. Correlation analysis showed that the MSV_RII_ of gray matter nuclei was correlated with cognitive function.

Our results showed that there has abnormal R_II_ iron deposition of gray matter nuclei in T2DM patients, after multiple comparison correction. There were significant differences in bilateral HCN, right PUT, right GP and left DN, while another ROI-based study found only PUT had significant difference (Li et al., 2020a). It suggests that regional iron analysis have higher diagnostic sensitivity than whole structural iron analysis. In addition, our results were partially consistent with another voxel-based study (Li et al., 2020b). Significant differences in HCN, PUT and GP on the right were found in both studies. Besides that, significant differences in left HCN and DN were also found. We thought that because of the uneven and relatively concentrated distribution of brain iron, the measurement of R_II_ iron is more sensitive, to find more areas of iron distribution difference.

A previous study on PD showed that the deposition of iron in the lateral SN pars compacta may be related to neuronal loss (Martin et al., 2008). Another study found that excessive iron accumulation was associated with region-specific advanced atrophy (Lee et al., 2013, 2015). Brain iron abnormally accumulates in a number of neurological disorders such as AD, PD leads to the neurotoxicity of oxidative stress which can promote neuron loss and degeneration, and may manifest as brain atrophy (Dixon et al., 2012; Ward et al., 2014; Rao et al., 2022; Yang et al., 2022). Lots of people believe that insulin resistance and diminished insulin signaling in the brain play a role in glucose hypometabolism (Freiherr et al., 2013; Ferreira et al., 2018). Since glucose is the primary source of energy for neurons, greatly reduced glucose metabolism is likely to affect both normal brain function and the processes of repairing and replacing critical cellular components for brain function when they are lost or damaged as a result of disease (Rao et al., 2022), and may also manifest as brain atrophy.

In this study, we found that iron deposition does exist in T2DM, and the iron deposition is accompanied by the reduction of the V_wh_, but the V_RII_ of T2DM was higher than that of HC group, thereby more V_RII_/V_wh_ regions with statistically significant differences than V_RII_ and V_wh_ regions were detected. This fully shows that the uneven iron distribution in the gray matter nuclei of T2DM patients, and the effect of secondary atrophy is offset by the fact that the area with high iron deposition is larger than HC, which is manifested by the increase of V_RII_. There was no significant correlation between MSV_RII_ and V_wh_ in T2DM group, maybe the changes of MSV_RII_ and V_wh_ are not synchronized, the shrinkage of the V_wh_ is caused by a combination of various factors, which needs further research.

Complicated changes of cerebral parenchymal microstructure were happened in T2DM patients, a single variable cannot be used to indicate the pathological changes. The results of this study also showed that compared with a single QSM parameter, the maximum AUC of combined parameters was 0.86, the sensitivity was 87.5%, the specificity was 75.9%, and a higher diagnostic value of comprehensive multiple parameters was found.

Brain iron level was strongly correlated with magnetic susceptibility *in vivo* and brain iron overload has recently been found associated with early cognitive impairment in animal and human models (Fujiwara et al., 2017; Wu et al., 2020). So far as we know, this is the first time to study the relationship between R_II_ iron and cognitive function in T2DM. We found that the MSV_RII_ of gray matter nuclei with significant differences were negatively correlated with cognitive impairment in T2DM after being corrected by multiple comparisons.

A prior research of iron deposition in T2DM patients found that the susceptibility values of the left PUT showed a close association with MMSE scores but not related to MoCA (Yang et al., 2018; Li et al., 2020a). However, we found the MSV_RII_ of the PUT in T2DM patients were significantly greater than HCs and not only had a close relationship with MMSE, but also negatively correlated with CVLT and MoCA scores. This result indicated that the MSV_RII_ reflect the cognitive function impairment more obviously than global susceptibility values due to the uneven distribution of iron in gray matter nuclei.

Globus pallidus exceeding all other gray nuclei has the highest iron concentration in the human brain (Langkammer et al., 2010; Modica et al., 2015). It is a target region of several neurodegenerative diseases with primary brain iron accumulation etiologies (Rouault, 2013), that had been confirmed in AD and multiple sclerosis (Wang et al., 2014; Fujiwara et al., 2017). Functionally, GP serves a range of cognitive and emotional functions (Schroder et al., 2013). In an MRI case-control study, multivariate regression analysis was used to identify the independent associations of brain iron overload and cognitive performance (Blasco et al., 2014). This result and our results strengthen the hypothesis that brains with regional high iron have a close relationship with cognitive decline. We found that the MSV_RII_ in the right GP had the highest correlation with List A LDFR. As List A LDFR is a reliable and quick test that is more sensitive to the deficit and gives more detailed information about verbal memory than a rough screening tool such as MMSE (Fox et al., 1998). We estimated that deficits in list learning and recall are hallmarks of T2DM.

Head of caudate nucleus not only regulate the movement of the body, but also has a certain impact on the sensory conduction process which is an important part of brain learning and memory system. We found that the increase of MSV_RII_ in the right HCN was significantly associated with the decrease of CVLT trials 1–5 Sum, List A SDFR, List A LDFR and MMSE scores. The change of MSV_RII_ in HCN can reflect cognitive impairment sensitively in T2DM.

## 5. Limitations

There are some limitations in this study. Firstly, the sample size was relatively small, a larger sample size is required to validate our findings. Secondly, manually outlining ROI will inevitably lead to small errors, we did not measure the iron content in the whole area, and R_II_ analysis greatly reduces the possibility of such errors. Thirdly, we only targeted gray matter nuclei rich in iron content, regions closely related to cognition, such as the hippocampus and amygdala, were not included. Fourth, only QSM quantitative measurement of STAGE was used for analysis, other quantitative images can also be used for further study in the future.

## 6. Conclusion

In T2DM patients, excessive and heterogeneous iron deposition as well as volume reduction occurs in deep gray nuclei. The MSV in high iron regions can better evaluate the distribution of iron, which is related to the decline of cognitive function. Further evaluations are needed to explore iron burden and volume atrophy in dementia pathology of T2DM in the future.

## Data availability statement

The original contributions presented in this study are included in this article/supplementary material, further inquiries can be directed to the corresponding authors.

## Ethics statement

The studies involving humans have been reviewed and approved by the First Affiliated Hospital of Dalian Medical University (PJ-KS-KY-2018-140). The patients/participants provided their written informed consent to participate in this study.

## Author contributions

RH and BG: conceptualization, study design, data curation and measurement, formal analysis, visualization, and writing—original draft. ST, YaL, YJ, WL, and YuL: data curation, software application, formal analysis, and investigation. QS: study design, data collection, supervision, and project administration. WW and YM: conceptualization, study design, funding acquisition, supervision, and writing—review and editing. All authors contributed to the article and approved the submitted version.
